# Liquid Cow’s Milk Consumption and Linear Growth Outcomes in Infancy and Childhood: A Systematic Review

**DOI:** 10.3390/nu18132083

**Published:** 2026-06-25

**Authors:** Jacksaint Saintila, Youmi Paz-Olivas

**Affiliations:** 1 Escuela de Posgrado, Universidad Peruana Unión, Lima 15464, Peru; 2 Escuela de Nutrición y Dietética, Universidad Femenina Sagrado Corazón (UNIFÉ), La Molina, Lima 15023, Peru; ypazo2050@gmail.com; 3 Área de Investigación, Instituto de Nutrición y Seguridad Alimentaria, Lima 15026, Peru

**Keywords:** cow’s milk, liquid milk, child growth, body height, infant, child, dietary intake, linear growth

## Abstract

**Background**: Linear growth during childhood is a key indicator of health status and child development, and liquid cow’s milk has been proposed as a potentially relevant dietary component for this outcome. In this systematic review, we aimed to synthesize the available evidence on the association between liquid cow’s milk consumption and linear growth outcomes in infants and children aged 6 months to 12 years. **Methods**: A systematic review was conducted in accordance with the Preferred Reporting Items for Systematic Reviews and Meta-Analyses (PRISMA) 2020 guidelines. Observational and experimental studies published in peer-reviewed journals, with no language restrictions, were included if they assessed habitual liquid cow’s milk consumption as the main exposure and reported linear growth outcomes such as height, growth velocity, or height-for-age z-scores. Searches were performed in PubMed (MEDLINE) and Scopus from database inception to 15 January 2026. Study selection, data extraction, and risk-of-bias assessment were carried out systematically. Due to methodological heterogeneity among the included studies, results were synthesized narratively. **Results**: Twelve studies conducted across diverse geographic and socioeconomic contexts were included. Most studies reported positive associations between liquid cow’s milk consumption and indicators of linear growth, including greater height, higher growth velocity, or improved height-for-age z-scores. Experimental studies showed significant increases in linear growth among children who received milk regularly, whereas some observational studies reported non-significant associations or results dependent on statistical adjustment. One study assessing complete cow’s milk exclusion observed deceleration in linear growth. Overall, the risk of bias was predominantly moderate. **Conclusions**: Habitual consumption of liquid cow’s milk during childhood appears to be predominantly associated with favorable linear growth outcomes, although variability exists according to study design, age at exposure, milk type, and exposure assessment. Further research using more robust designs is warranted to clarify the magnitude of the association, potential mechanisms, and implications for weight-related outcomes.

## 1. Introduction

Linear growth during childhood, commonly assessed using height and height-for-age z-scores (HAZ) [1], represents one of the most robust and integrative indicators of child health and development [2]. Unlike other anthropometric markers that are more sensitive to acute fluctuations, linear growth reflects the cumulative interaction of nutrition [3], health status [3,4], the social environment [5], and biological conditions during critical periods of development. For this reason, child stature has been widely used as a proxy marker of child well-being [6], adequate physical development [1,2] and, in the long term, population human capital [6]. Suboptimal linear growth is associated with adverse consequences that extend beyond attained height, including increased susceptibility to infections [7], a higher risk of childhood morbidity and mortality [8], and impairments in cognitive development [7,9], school performance [9] and economic productivity later in life [10].

Despite global improvements in nutritional indicators and the progress of the nutrition transition in many countries, linear growth remains a priority outcome in child public health [1,2,3]. In settings where chronic undernutrition, overweight, and obesity coexist [11,12], height-for-age continues to provide critical information on structural inequalities, dietary quality [13,14] and exposure to adverse early-life determinants [3,4]. In this context, identifying dietary factors that may be associated with linear growth is essential to inform policies and intervention strategies aimed at promoting healthy and equitable child development.

Dietary intake represents one of the most relevant modifiable determinants of linear growth during childhood, as it directly influences the availability of energy [15], macronutrients [16], and micronutrients [17] required for tissue synthesis and skeletal development [16,17]. Adequate and sustained intake is particularly critical during sensitive growth periods, in which even moderate nutritional deficits may translate into cumulative impairments in height-for-age [18]. Within this context, liquid milk has received particular attention as dietary exposure [19,20], given its characteristic nutritional profile and its frequent consumption during childhood across diverse cultural [21] and socioeconomic settings [22]. Milk provides high-biological-quality proteins, energy, and essential micronutrients for growth and bone mineralization, such as calcium and phosphorus, and in some contexts, vitamin D [23]. This has motivated the evaluation of its potential association with indicators of linear growth in multiple observational [24,25,26] and experimental studies [27,28,29,30].

Indeed, milk consumption has been proposed to influence linear growth through mechanisms related to its nutritional matrix [31,32], its protein density [33] and the stimulation of hormonal pathways involved in growth, such as the insulin-like growth factor 1 (IGF-I) axis [31,32,34]. This biological plausibility provides a theoretical basis for considering milk as a relevant dietary exposure in the study of child growth and underscores the need to systematically synthesize the available evidence regarding its relationship with linear growth.

Nevertheless, the existing evidence presents limitations that hinder the derivation of clear and consistent conclusions. Previous studies have varied widely in the age groups included, the outcomes assessed, the methods used to measure milk consumption, and the study designs employed, all of which have contributed to heterogeneous findings. In particular, a substantial proportion of investigations have prioritized weight-related indicators, such as body weight or body mass index [35,36,37,38,39], whereas linear growth has not always been addressed as a primary outcome, despite its greater relevance as a marker of long-term child development. In addition, several reviews have grouped different dairy products or broad dietary patterns, without systematically distinguishing liquid milk consumption as a specific exposure or explicitly considering the developmental stage during which such exposure occurs.

In light of this heterogeneity, a systematic review focused on liquid cow’s milk consumption and linear growth during infancy and childhood is warranted. Therefore, the objective of the present systematic review was to critically synthesize the available scientific evidence on the association between habitual liquid cow’s milk consumption and linear growth outcomes in children aged 6 months to 12 years, integrating observational and experimental studies conducted across diverse geographic and socioeconomic contexts.

## 2. Materials and Methods

### 2.1. Study Design

A systematic review of the literature was conducted in accordance with the recommendations of the PRISMA 2020 statement (Preferred Reporting Items for Systematic Reviews and Meta-Analyses) [40,41]. The aim of the review was to synthesize the available scientific evidence on the relationship between habitual liquid cow’s milk consumption and linear growth outcomes in children aged 6 months to 12 years.

**Figure 1:**
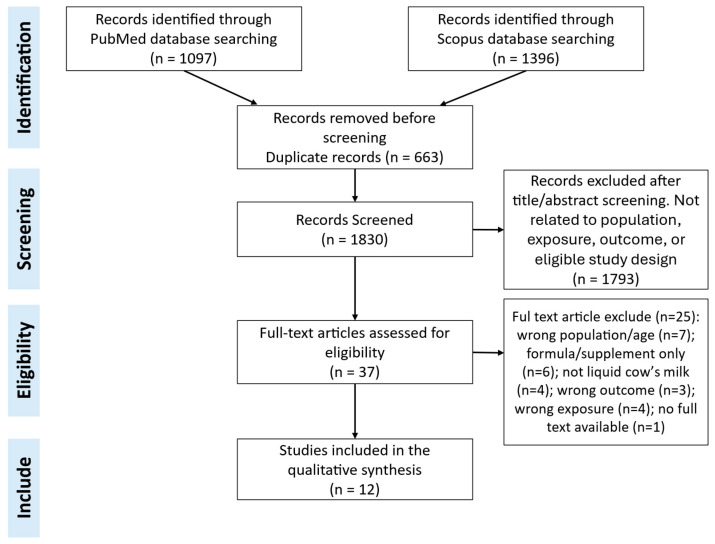
Flow diagram of the study identification, screening, and eligibility process for studies included in the systematic review, in accordance with the PRISMA statement [40,41].

Eligible designs were defined a priori and included observational studies (cohort, longitudinal, and cross-sectional studies), nested case–control studies, and experimental studies or controlled interventions with at least 6 months of exposure. Thus, no design filters were applied during the initial database search in order to maximize sensitivity; however, study design was evaluated during full-text eligibility assessment according to the predefined inclusion criteria. The processes of study identification, screening, eligibility assessment, and final inclusion are summarized in a PRISMA-compliant flow diagram (Figure 1).

### 2.2. Inclusion and Exclusion Criteria

Study selection was based on predefined criteria structured according to the Population, Exposure, Outcome, and Comparator (PICO) framework.

Studies conducted in pediatric populations were included, specifically those involving children aged 6 months to 12 years, provided that exposure to liquid cow’s milk consumption and the assessment of linear growth outcomes occurred within this age range, even if participant follow-up extended beyond 12 years of age. Eligible studies were required to evaluate habitual consumption of liquid cow’s milk as the main exposure. Studies that included milk together with other dairy products were eligible only when liquid milk was analyzed separately or clearly represented the primary exposure. Studies had to report linear growth outcomes, such as height, growth velocity, length, or standardized indicators including HAZ or length-for-age z-scores (LAZ), and to analyze the association between cow’s milk consumption and these outcomes.

Observational studies (cohort, longitudinal, and cross-sectional studies), nested case–control studies, and experimental trials were considered, provided that the exposure corresponded to habitual liquid cow’s milk consumption rather than short-term interventions (<6 months in duration). Studies conducted in any country and socioeconomic context were included, with no language restrictions, as long as they were published in peer-reviewed scientific journals.

Some intervention studies included in the review evaluated the consumption of fortified cow’s milk (e.g., with calcium, with or without vitamin D) [27,28]. These interventions were considered eligible because liquid cow’s milk was the main exposure. However, it is acknowledged that the observed effects on linear growth may reflect both the milk matrix and the contribution of added micronutrients, rather than the effect of cow’s milk alone.

Studies that exclusively evaluated other dairy products, such as yogurt or cheese, without a specific analysis of liquid cow’s milk consumption were excluded. Likewise, investigations focused exclusively on infant formulas used for therapeutic purposes or as supplements were excluded, as were studies that reported only outcomes related to body weight, body mass index, or other indicators not directly related to linear growth. Narrative or systematic reviews, editorials, letters to the editor, conference abstracts, and animal studies were also excluded.

### 2.3. Search Strategy and Study Selection

The systematic literature search was conducted in PubMed (MEDLINE) and Scopus from database inception to 15 January 2026. The search strategy was designed to identify studies that simultaneously addressed pediatric populations, cow’s milk consumption, and linear growth outcomes. Full search strings are provided in Supplementary Table S1.

Combinations of terms related to childhood, cow’s milk exposure, and linear growth were used with Boolean operators (“AND”, “OR”). No filters for study design were applied during the initial search phase in order to ensure broad retrieval of potentially relevant literature; study design restrictions were applied during full-text screening based on the predefined eligibility criteria.

All identified records were exported to reference management software, where duplicates were removed prior to screening. Titles and abstracts were then assessed for initial eligibility. Potentially relevant studies underwent full-text evaluation. Full-text exclusions were classified according to the following reasons: wrong population or age range, not liquid cow’s milk, formula or supplement only, wrong exposure, wrong outcome, review/editorial/commentary, other ineligibility reason, or no full text available. Discrepancies were resolved through review and consensus.

### 2.4. Data Extraction

Data extraction was performed using a previously developed standardized form. For each included study, information was collected on general study characteristics (author, year of publication, and country), methodological design, sample size, and characteristics of the study population.

Detailed information on the exposure was extracted, including the type of milk assessed, fat content when reported, fortification status, lactose-free or added-sugar status when available, method used to measure consumption, comparator or reference group, and dietary assessment tools. With respect to outcomes, the reported linear growth indicators, anthropometric measurement methods, growth reference standards, effect estimates, and covariates included in adjusted models were recorded when available.

When relevant data were not clearly specified in the article, they were recorded as “not reported.” Given the heterogeneity observed in study designs, exposure measures, comparators, and evaluated outcomes, the results were synthesized narratively, with the presentation of findings structured according to the main linear growth outcomes reported. Comparator/reference groups and main adjusted covariates are summarized in Supplementary Table S3.

### 2.5. Risk of Bias Assessment

The methodological quality and risk of bias of the included studies were assessed using the Newcastle–Ottawa Scale (NOS), in accordance with the recommendations of the Cochrane Handbook for the evaluation of non-randomized studies [42,43,44]. This tool is widely used to appraise the methodological quality of observational studies and allows for a structured assessment based on key domains of study design [45]. For randomized and controlled intervention studies, the NOS was used as a structured framework and interpreted narratively, considering trial-specific features such as group comparability, supervision of intake, blinding, and potential co-intervention effects related to micronutrient fortification.

For cohort studies, the NOS evaluates three main domains: selection of participants (maximum of 4 points), comparability between groups (maximum of 2 points), and outcome assessment (maximum of 3 points). For case–control studies, the domains of selection (maximum of 4 points), comparability (maximum of 2 points), and exposure assessment (maximum of 3 points) were considered. Each study was scored independently according to these criteria, yielding a maximum total score of 9 points.

The results were interpreted using commonly accepted cut-off points, classifying studies as having a low risk of bias (7–9 points), moderate risk of bias (4–6 points), or high risk of bias (0–3 points) [46]. Domain-level scores and total NOS scores for each study are presented in Supplementary Table S2 and were considered in the narrative synthesis and interpretation of the evidence.

## 3. Results

### 3.1. Study Characteristics

The systematic review included 12 studies conducted across diverse geographic and socioeconomic contexts, including the United States, Europe, Asia, and Africa [24,26,47] (Table 1). The methodological designs primarily comprised longitudinal and birth cohort studies, as well as controlled trials and school-based interventions, with sample sizes varying widely from 122 to 8950 participants [24,34]. The included studies covered infancy and childhood; however, in all cases, exposure to liquid cow’s milk consumption and the assessment of linear growth occurred between 6 months and 12 years of age. Some studies extended follow-up beyond this age range to evaluate long-term growth trajectories [25,48,49].

Most studies included both boys and girls, with female representation close to 50%, whereas two investigations were conducted exclusively in female populations [27,48]. The follow-up period ranged from one year to up to 17 years, with some studies employing monthly or repeated assessments during early stages of growth [26,50].

Linear growth was assessed using different reference standards, depending on the study context. The World Health Organization (WHO) Child Growth Standards were the most commonly used, particularly in studies conducted in low- and middle-income countries [34,51], whereas studies carried out in the United States used the CDC 2000 growth charts [24,48]. Other studies relied on national reference standards, such as those from China, Finland, and the United Kingdom, and some older trials did not explicitly report the growth standard applied [28,29]. Comparator/reference groups and adjusted covariates are provided in Supplementary Table S3.

### 3.2. Exposure to Milk and Linear Growth Outcomes

Table 2 describes the methods used to assess exposure to liquid cow’s milk consumption and linear growth outcomes in the included studies. Exposure was measured using food frequency questionnaires, short-term dietary records, or supervised school-based interventions, with variability in quantification methods across studies. In observational studies, liquid cow’s milk consumption was estimated from questionnaires completed by caregivers or participants and was expressed as frequency, daily servings, or average volume consumed [24,25,26]. Some studies categorized exposure according to volume or frequency [34,49,51], whereas others assessed milk together with dairy products or animal-source foods using food frequency questionnaires or repeated dietary recalls [48,50].

In controlled trials and intervention studies, exposure consisted of daily provision of liquid cow’s milk, in some cases fortified with calcium and vitamin D, under supervised school-based conditions [27,28,29]. In one study, exposure was defined as the complete elimination of cow’s milk as part of an exclusion diet [47]. Linear growth outcomes were assessed using standardized anthropometric measurements, including height, length, growth velocity, and height- or length-for-age z-scores, based on international or national reference standards, such as the WHO growth standards, CDC growth charts, or country-specific references [24,34,49].

### 3.3. Summary of Findings and Risk of Bias

Table 3 shows that most included studies reported a positive association between liquid cow’s milk consumption and linear growth outcomes in pediatric populations. Large observational studies indicated that higher milk intake was associated with greater height or more favorable growth trajectories during childhood and adolescence [24,25,48,49]. School-based intervention studies also demonstrated significant increases in height gain, particularly in those using fortified milk; however, in these cases, the observed effects may be attributable to both the milk matrix and added micronutrients [27,28].

In a birth cohort study, milk consumption was associated with higher IGF-I concentrations and better indicators of linear growth at two years of age [34], whereas in low-resource settings, milk intake as part of animal-source foods was related to higher height-for-age scores [26,50]. However, not all studies observed statistically significant associations after adjustment for confounding variables, as shown in a study conducted during the COVID-19 pandemic in Indonesia, where the association between milk consumption and growth was attenuated after adjustment [51].

**Table 1:** Characteristics of the studies included in the systematic review (*n* = 12).

Ref.	Author (Year)	Country	Study Design	Sample Size (n)	Age	Sex Distribution	Follow-Up Period	Growth Reference Standard
[51]	Setyawati et al. (2022)	Indonesia	Prospective cohort	565	<5 years	Both sexes (55.2% female)	2 years (2019–2021)	WHO growth standards
[25]	Marshall et al. (2018)	USA	Birth cohort (17-year follow-up)	717	2–17 years	Both sexes (49% female)	Birth to 17 years	Not reported
[34]	Wiley et al. (2018)	India	Birth cohort	122	Birth–2 years	Both sexes (47% female)	Birth to 2 years	WHO Child Growth Standards
[47]	Tuokkola et al. (2017)	Finland	Nested case–control	560	1–5 years	Both sexes (boys and girls)	Birth to 5 years (annual follow-up)	Finnish national growth standards
[26]	Mosites et al. (2017)	Kenya	Longitudinal cohort	874	6–59 months	Both sexes (52.9% female)	1 year (monthly follow-up)	WHO growth standards
[50]	Muslimatun & Wiradnyani (2016)	Indonesia	Longitudinal study	210	1–5 years	Both sexes (56.4% female)	1 year (four assessments at 16–20-week intervals)	WHO growth standards
[24]	DeBoer et al. (2015)	USA	Longitudinal cohort	8950	4–5 years	Both sexes (≈49% female)	1 year (ages 4 to 5 years)	CDC growth charts (2000)
[49]	Hopkins et al. (2015)	UK	Birth cohort	1112	8 months	Both sexes (≈51% male, 49% female)	Birth to 10 years	UK 1990 growth reference
[48]	Berkey et al. (2009)	USA	Prospective cohort	5101	9–14 years	Girls only (100% female)	Up to ~8 years of follow-up	CDC growth charts (2000)
[28]	Du et al. (2004)	China	Randomized controlled trial	681	10–12 years	Both sexes (boys and girls)	2 years	Not reported
[27]	Zhang et al. (2003)	China	Controlled intervention	710	10–12 years	Girls only (100% female)	2 years	Chinese national growth standards
[29]	Baker et al. (1980)	UK	Randomized controlled trial	581	7–8 years	Both sexes (boys and girls)	~21.5 months (six school terms)	Not reported

Note: WHO, World Health Organization; CDC, Centers for Disease Control and Prevention. Detailed comparator/reference groups are provided in Supplementary Table S3.

**Table 2:** Assessment of liquid cow’s milk exposure and linear growth outcomes across included studies.

Ref.	Study (Author, Year)	Type of Milk Exposure	Exposure Assessment	Outcome Assessment	Reference/Comparator Group	Overall Direction of Association
[24]	DeBoer et al., 2015	Cow’s milk intake (liquid), quantified as servings/day (1 serving = 236 mL); categories: 0, <1, 1, 2, 3, ≥4 servings/day	Caregiver-reported 7-day milk intake frequency questionnaire; frequency converted to daily servings; type of milk recorded	Height and HAZ measured using CDC growth standards at ages 4 and 5 years	Lower milk intake categories among milk drinkers	Positive association with height and HAZ, persisting longitudinally
[48]	Berkey et al., 2009	Intake of milk, yogurt, and cheese assessed separately	Semi-quantitative FFQ, self-administered; repeated annually	Annual height gain and attained adult height derived from repeated height measurements	Lower intake categories of milk, yogurt, cheese, dairy protein, or dairy calcium	Positive for milk and yogurt; no consistent association for cheese
[25]	Marshall et al., 2018	Cow’s milk intake (liquid), quantified as mean daily volume across childhood and adolescence; modeled per 8 oz (236 mL) increment	Validated beverage frequency questionnaires administered repeatedly at 3–6-month intervals; milk intake averaged longitudinally	Repeated height measurements (cm) obtained using stadiometer during clinic visits from childhood to adolescence	Lower mean daily milk intake; modeled per 8 oz increment	Positive longitudinal association between higher milk intake and greater height
[28]	Du et al., 2004	Calcium- and vitamin D–fortified cow’s milk intervention (≈144 mL/day)	School-based randomized controlled milk intervention	Height and sitting height measured using standardized anthropometric procedures	School control group without fortified milk	Positive association with linear growth
[34]	Wiley et al., 2018	Cow’s milk intake (liquid), quantified as daily volume (mL/day); categorized as <250, 250–500, and >500 mL/day	Semi-quantitative food frequency questionnaire and maternal interview at 2 years; milk intake classified in 250 mL increments	Length and length-for-age z-score (LAZ) assessed using WHO growth standards at 2 years	<250 mL/day milk intake	Positive association between higher milk intake and greater length/linear growth
[29]	Baker et al., 1980	Cow’s milk supplementation (fixed daily volume: 190 mL/day)	School-based randomized intervention with supervised milk intake	Height gain assessed using standardized height measurements	Control/no school milk supplementation	Positive (small but statistically significant increase in height gain)
[26]	Mosites et al., 2017	Cow’s milk consumption assessed by feeding frequency	Caregiver-reported 3-day feeding frequency questionnaire, repeated monthly	Monthly height gain measured using standardized length/height measurements (WHO standards)	Lower/no cow’s milk feeding frequency	Positive association between higher milk consumption frequency and monthly height gain
[27]	Zhang et al., 2003	Calcium- and vitamin D–fortified cow’s milk intervention (144 mL/day)	School-based controlled intervention with daily fortified milk intake	Height and knee height measured using standardized anthropometric procedures	School control group without fortified milk	Positive association with linear
[51]	Setyawati et al., 2022	Milk consumption frequency (>1 time/week vs. ≤1 time/week); includes liquid, powdered, or formula milk (sweetened condensed milk excluded)	Parent-reported food consumption frequency collected via structured questionnaire during the COVID-19 pandemic (2021)	HAZ categories assessed in 2019 and 2021 using standardized height measurements	≤1 time/week milk consumption	Positive association with improvement in HAZ category (not statistically significant after adjustment)
[47]	Tuokkola et al., 2017	Cow’s milk elimination diet (complete exclusion vs. consumption), compared with wheat/barley/rye elimination and controls	Parent-reported elimination diet due to food allergy; assessed at 6, 12, and 24 months and via structured questionnaire at 3 years; supported by 3-day food records	HAZ and growth velocity assessed using national growth standards, followed annually up to 5 years	Cow’s milk consumers and controls	Negative association: cow’s milk elimination associated with growth deceleration
[50]	Muslimatun & Wiradnyani, 2016	Milk and dairy intake as part of animal-source food consumption	Repeated 24 h dietary recalls collected longitudinally	HAZ assessed using WHO growth standards	Lower/no milk and dairy or animal-source food intake	No significant association with linear growth
[49]	Hopkins et al., 2015	Cow’s milk and formula intake in late infancy, categorized by daily volume (<600 vs. ≥600 mL/day)	Prospective 3-day unweighed food records at 8 months; milk type and daily volume quantified according to UK infant-feeding recommendations	Height standard deviation score (Height SDS) derived from repeated standardized measurements from birth to 10 years	Breast milk reference and lower-volume milk/formula groups	Positive association between high-volume milk intake and greater height gain

Note: HAZ, height-for-age z-score; LAZ, length-for-age z-score; SDS, standard deviation score; FFQ, food frequency questionnaire. Adjusted covariates and comparator/reference groups are provided in Supplementary Table S3.

**Table 3:** Summary of findings and Newcastle–Ottawa Scale (NOS) risk-of-bias assessment of the included studies.

Ref.	Study (Author, Year)	Key Findings (Observation)	Authors’ Conclusions	NOS Score/Risk of Bias *
[24]	DeBoer et al., 2015	Higher cow’s milk intake was associated with greater height in preschool children, independent of BMI.	Milk consumption may support linear growth during early childhood.	6/9, Moderate
[48]	Berkey et al., 2009	Higher milk and yogurt intake were associated with greater linear growth and taller adult height; dairy protein showed the strongest association.	Milk and dairy intake were positively associated with linear growth.	6/9, Moderate
[25]	Marshall et al., 2018	Longitudinal milk intake was positively associated with height trajectories up to adolescence.	Sustained milk consumption supports long-term linear growth.	8/9, Low
[28]	Du et al., 2004	Children receiving fortified milk showed significantly greater height gain compared to controls.	School-based milk interventions may enhance growth, although micronutrient fortification may contribute.	5/9, Moderate
[34]	Wiley et al., 2018	Milk intake was positively associated with height-for-age and length, accompanied by higher IGF-I concentrations at 2 years.	Milk consumption may support linear growth through IGF-I–related pathways	6/9, Moderate
[29]	Baker et al., 1980	Milk supplementation resulted in increased height increments in school-aged children.	Regular milk intake may promote growth in childhood.	3/9, High
[26]	Mosites et al., 2017	Milk consumption was associated with higher height-for-age z-scores in young children.	Animal-source foods, including milk, support linear growth.	6/9, Moderate
[27]	Zhang et al., 2003	Fortified milk intake significantly increased height velocity in children.	Milk-based interventions improve growth, potentially through combined milk matrix and micronutrients.	5/9, Moderate
[51]	Setyawati et al., 2022	Milk consumption > 1 time/week was associated with improvement in height-for-age category, although the association was attenuated after adjustment.	Milk consumption may contribute to improved growth status, though effects appear modest after adjustment.	5/9, Moderate
[47]	Tuokkola et al., 2017	Cow’s milk elimination was associated with growth deceleration and lower height compared with children consuming cow’s milk.	Avoidance of cow’s milk in early childhood may compromise linear growth.	7/9, Low
[50]	Muslimatun & Wiradnyani, 2016	Diets including milk and animal-source foods were associated with improved micronutrient adequacy, but milk intake was not independently associated with height-for-age	Milk and other animal-source foods may contribute to child nutrition, although evidence for an independent effect on linear growth was limited.	5/9, Moderate
[49]	Hopkins et al., 2015	Milk intake was associated with increased height gain in children.	Higher milk intake was associated with greater height gain during childhood.	6/9, Moderate

Note. * Risk of bias was assessed using the Newcastle–Ottawa Scale (NOS) and interpreted narratively based on study design, exposure assessment, outcome measurement, and control of confounders. Detailed domain-level scoring is provided in Supplementary Table S2.

In contrast, the only study that evaluated complete elimination of cow’s milk reported a negative association, showing growth deceleration and lower height values compared with children who consumed cow’s milk [47].

Most studies were classified as having moderate risk of bias, mainly due to limitations inherent to dietary assessment methods and control of confounding factors. Two studies were rated as low risk of bias, whereas one older trial was classified as high risk of bias [25,29,47]. Numerical NOS scores are reported in Table 3 and detailed domain-level scores are provided in Supplementary Table S2.

## 4. Discussion

The findings of this systematic review indicate that habitual consumption of liquid cow’s milk is predominantly associated with favorable linear growth outcomes during childhood, although the magnitude and consistency of this association vary according to context, study design, age at exposure, milk type, and exposure assessment methods. Most included studies, particularly longitudinal studies with large sample sizes, reported positive associations between higher milk intake and greater height, growth velocity, or height-for-age z-scores in both preschool-aged and school-aged children [24,25]. This pattern suggests that the relationship between milk consumption and linear growth may persist across different stages of child development. In contrast, some studies reported non-significant or model-dependent associations, particularly in settings where exposure was assessed using less precise instruments or where multiple nutritional determinants of growth coexisted [50,51]. Nevertheless, in these studies, the lack of statistically significant associations did not contradict the overall pattern observed in investigations with more accurate exposure measurements.

Consistently, the experimental studies included in this review demonstrated significant increases in linear growth among children who received milk regularly in school-based settings compared with control groups [27,28,29]. Although several of these interventions used fortified milk, their findings are in line with the observational evidence and reinforce the biological plausibility of a positive association between exposure to liquid milk and linear growth indicators. Therefore, the convergence of results from observational and experimental studies conducted across diverse geographic and population contexts strengthens the synthesized evidence and reduces the likelihood that the observed associations are explained solely by confounding factors or biases inherent to a single study design, particularly in longitudinal studies with repeated outcome measurements.

The findings observed in this review may be mediated by multiple nutritional and hormonal mechanisms associated with milk consumption during childhood. In particular, stimulation of the insulin-like growth factor 1 (IGF-I) axis has been proposed as one of the most consistent mechanisms, given its central role in the regulation of linear growth. In this regard, the study by Wiley et al. [34] showed that higher milk intake at two years of age was associated with higher serum IGF-I concentrations and better length-for-age indicators, which is consistent with the hypothesis that the nutritional matrix of milk may play a role in early linear growth. In addition, milk provides high-biological-quality proteins, energy, and key micronutrients essential for bone growth, which may jointly contribute to the observed outcomes across different contexts [24,25]. In school-based trials, increases in height may reflect both the regular provision of liquid milk and, in some cases, the additional contribution of micronutrients such as calcium and vitamin D [27,28].

Nevertheless, it is important to note that most of the included studies were not designed to directly assess the underlying physiological mechanisms; therefore, the proposed explanations should be interpreted as biologically plausible hypotheses rather than as confirmed causal relationships. Consequently, although the available evidence suggests potential hormonal and nutritional pathways that may explain the association between milk consumption and linear growth, specific studies integrating detailed dietary assessments with growth-related biomarkers are needed to clarify these mechanisms more precisely.

Despite the predominance of positive associations, heterogeneity was observed in the magnitude and statistical significance of results. This heterogeneity may be explained by differences in age at exposure, baseline nutritional status, follow-up duration, and exposure definition. The age at which exposure was assessed ranged from late infancy to middle childhood [34,48], while follow-up varied from short-term assessments to extended trajectories [25,49]. Cow’s milk exposure was also operationalized heterogeneously—as frequency, daily volume, standardized servings, fortified milk provision, or part of broader dietary patterns—introducing variability into dietary exposure estimation [24,26,51].

A distinctive finding was reported by Tuokkola et al. [47], who observed that complete elimination of cow’s milk during early childhood was associated with slower linear growth compared with children who consumed cow’s milk. Unlike studies assessing different levels of intake, this study evaluated a dietary exclusion scenario, reporting lower height-for-age scores and reduced growth velocity during follow-up. However, because cow’s milk elimination was clinically indicated due to food allergy, these findings should be interpreted cautiously and should not be directly extrapolated to healthy children without a medical indication for milk exclusion.

Another important source of heterogeneity is milk composition. Few studies reported sufficient detail on fat content, added sugar, lactose-free status, or whether milk was whole, reduced-fat, skimmed, fortified, or unfortified. For example, DeBoer et al. [24] recorded milk type, whereas many studies did not consistently report fat composition. Because whole milk may provide more energy in younger children, whereas reduced-fat or skimmed milk may be preferred in older children or children at risk of excessive weight gain, the lack of consistent reporting prevents firm conclusions regarding whether milk fat content modifies the association with linear growth [52]. Similarly, the available evidence was insufficient to synthesize potential differences according to lactose-free or sweetened milk products.

Weight-related outcomes should also be considered when interpreting the findings. Although this review focused on linear growth, some included studies reported BMI, weight gain, or weight-for-height outcomes [24,48,49]. For example, higher milk intake may be associated with greater overall growth, including both linear and ponderal growth [25], and accelerated growth velocity has been linked to later adiposity risk in some contexts [53,54]. Therefore, the findings should not be interpreted as supporting high-volume cow’s milk intake without consideration of total diet quality, age, adiposity status, and current pediatric nutrition guidance.

The results should not be generalized directly to non-cow milk beverages. Goat milk and goat-milk-based infant formulas were outside the eligibility criteria of this review, as were plant-based beverages and therapeutic formulas. Existing evidence on goat-milk-based formulas addresses infant growth and safety compared with cow-milk-based formulas, but this differs from habitual liquid cow’s milk consumption in children aged 6 months to 12 years [55]. Dedicated reviews are needed to evaluate whether non-cow milk beverages have comparable associations with linear growth [56].

### 4.1. Risk of Bias and Quality of the Evidence

Most included studies were classified as having moderate risk of bias, mainly due to limitations in exposure assessment and potential residual confounding inherent to observational designs [24,26,48]. Dietary exposure was predominantly assessed using self-reported instruments, which are susceptible to recall bias and imprecision. Although linear growth outcomes were generally measured using standardized anthropometric methods, not all studies provided detailed descriptions of calibration or evaluator standardization. In controlled trials, the lack of blinding and the use of fortified milk limit attribution of observed effects exclusively to liquid cow’s milk. Nevertheless, consistency in the direction of associations, particularly in longitudinal studies with repeated outcome measurements, suggests that findings cannot be attributed solely to methodological bias.

### 4.2. Strengths and Limitations

This review has several strengths, including the use of PRISMA 2020 guidance [40,41], predefined eligibility criteria focused on linear growth, inclusion of both observational and experimental evidence, and structured assessment of risk of bias. The review also distinguished liquid cow’s milk from other dairy products and formulas, thereby improving exposure specificity. Limitations include heterogeneity in exposure measurement, differences in growth reference standards, limited reporting of milk composition, and the narrative nature of the synthesis. In addition, although one author is employed by Gloria Foods S.A., this affiliation did not influence the search strategy, eligibility criteria, data extraction, analysis, or interpretation of findings; transparency regarding this potential conflict was maintained throughout the manuscript.

### 4.3. Implications and Future Research Directions

The findings of this review have implications for nutritional research and child public health, suggesting that liquid cow’s milk consumption during childhood may be associated with favorable linear growth outcomes across diverse settings. Future studies should use robust longitudinal designs, precise and repeated dietary assessments, and growth-related biomarkers. It is also important to distinguish unfortified from fortified cow’s milk, evaluate the role of milk fat content and added sugars, and examine whether associations differ according to age, baseline nutritional status, and adiposity risk. Studies assessing non-cow milk beverages and clinically indicated milk exclusion diets are also needed to guide evidence-based dietary recommendations.

## 5. Conclusions

This systematic review synthesized evidence from 12 studies evaluating the association between habitual liquid cow’s milk consumption and linear growth outcomes in children aged 6 months to 12 years. Overall, most studies reported positive associations with height, growth velocity, or height-for-age z-scores, and school-based interventions generally showed greater height gain among children receiving milk. However, the magnitude and statistical significance of findings varied according to study design, age at exposure, exposure assessment, milk type, and adjustment for confounders. Evidence from fortified milk interventions suggests potential benefit, but it does not allow complete isolation of the effect attributable to cow’s milk itself. These findings support the relevance of liquid cow’s milk as a dietary exposure related to child linear growth, while emphasizing the need for future studies that distinguish milk composition, consider weight-related outcomes, and use more precise longitudinal assessments.

## Data Availability

No new data were created or analyzed in this study.

## Supplementary Materials

The following supporting information can be downloaded at: https://www.mdpi.com/article/10.3390/nu18132083/s1, Table S1: Search strategies used in PubMed/MEDLINE and Scopus; Table S2: Newcastle-Ottawa Scale (NOS) domain-level scoring and risk-of-bias classification; Table S3: Comparator/reference groups and main covariates adjusted or considered in the included studies.

## References

[1] Watson K.M., Dasiewicz A.S., Bassani D.G., Chen C.Y., Qamar H., Roth D.E. (2025). Quantifying child growth effects using height-age instead of height-for-age z-scores in a meta-analysis of small-quantity lipid-based nutrient supplement trials. Sci. Rep..

[2] Foote J.M. (2014). Optimizing linear growth measurement in children. J. Pediatr. Health Care.

[3] Inzaghi E., Pampanini V., Deodati A., Cianfarani S. (2022). The Effects of Nutrition on Linear Growth. Nutrients.

[4] Millward D.J. (2017). Nutrition, infection and stunting: The roles of deficiencies of individual nutrients and foods, and of inflammation, as determinants of reduced linear growth of children. Nutr. Res. Rev..

[5] Amugsi D.A., Dimbuene Z.T., Kimani-Murage E.W. (2020). Socio-demographic factors associated with normal linear growth among pre-school children living in better-off households: A multi-country analysis of nationally representative data. PLoS ONE.

[6] Esquivel-Lauzurique M., González-Fernández C., Rubén-Quesada M., Machado-Lubián M.D.C., Tamayo-Pérez V. (2019). Cuban Experience Using Growth and Development as a Positive Indicator of Child Health. MEDICC Rev..

[7] de Onis M., Branca F. (2016). Childhood stunting: A global perspective. Matern. Child Nutr..

[8] Mertens A., Benjamin-Chung J., Colford J.M., Coyle J., Van der Laan M.J., Hubbard A.E., Rosete S., Malenica I., Hejazi N., Sofrygin O. (2023). Causes and consequences of child growth faltering in low-resource settings. Nature.

[9] Bundy D.A., De Silva N., Horton S., Patton G.C., Schultz L., Jamison D.T. (2017). Evidence of Impact of Interventions on Growth and Development during Early and Middle Childhood. Disease Control Priorities: Child and Adolescent Health and Development.

[10] Sitorus N.L. (2024). The Significance of Tackling Stunting for The Economic Prosperity of a Nation—A Narrative Review. J. Indones. Spec. Nutr..

[11] Bermúdez J.N., Ayala D., Herrán O.F. (2020). Nutrition gap in children, urban-rural: The key education and food. Colombia, 2015. Rev. Saude Publica.

[12] Rabiei S., Ebrahimof S., Rasekhi H., Amini M., Ghodsi D., Yari Z., Abdollahi Z., Minaie M., Nikooyeh B., Neyestani T.R. (2024). Exploring the determinants of malnutrition in 2–5 year Iranian children using structural equation modeling: National food and nutrition surveillance. BMC Public Health.

[13] Rotella R., Morales-Suarez-Varela M., Llopis-Gonzalez A., Soriano J.M. (2025). A Nutritional and Anthropometric Analysis of the Double Burden of Malnutrition in Children Under Two in Madagascar. Children.

[14] Dinku A.M., Mekonnen T.C., Adilu G.S. (2020). Child dietary diversity and food (in)security as a potential correlate of child anthropometric indices in the context of urban food system in the cases of north-central Ethiopia. J. Health Popul. Nutr..

[15] Zhang W., Murray R., Barkley G., Singh N., Rhoads R.P., Stahl C.H. (2019). Caloric Intake Affects Neonatal Bone Development and Energy Metabolism. FASEB J..

[16] Savarino G., Corsello A., Corsello G. (2021). Macronutrient balance and micronutrient amounts through growth and development. Ital. J. Pediatr..

[17] Hasan S., Naseer S., Zamzam M., Mohilldean H., Van Wagoner C., Hasan A., Saleh E.S., Uhley V., Kamel-ElSayed S. (2024). Nutrient and Hormonal Effects on Long Bone Growth in Healthy and Obese Children: A Literature Review. Children.

[18] Zhang Z., Li F., Hannon B.A., Hustead D.S., Aw M.M., Liu Z., Chuah K.A., Low Y.L., Huynh D.T. (2021). Effect of Oral Nutritional Supplementation on Growth in Children with Undernutrition: A Systematic Review and Meta-Analysis. Nutrients.

[19] Grenov B., Larnkjær A., Mølgaard C., Michaelsen K.F. (2020). Role of Milk and Dairy Products in Growth of the Child. Nestle Nutr. Inst. Workshop Ser..

[20] Grenov B., Michaelsen K.F. (2018). Growth Components of Cow’s Milk: Emphasis on Effects in Undernourished Children. Food Nutr. Bull..

[21] Neves P.A., Vaz J.S., Maia F.S., Baker P., Gatica-Domínguez G., Piwoz E., Rollins N., Victora C.G. (2021). Rates and time trends in the consumption of breastmilk, formula, and animal milk by children younger than 2 years from 2000 to 2019: Analysis of 113 countries. Lancet Child Adolesc. Health.

[22] Neves P.A., Barros A.J., Baker P., Piwoz E., Santos T.M., Gatica-Domínguez G., Vaz J.S., Rollins N., Victora C.G. (2022). Consumption of breast milk, formula and other non-human milk by children aged under 2 years: Analysis of eighty-six low- and middle-income countries. Public Health Nutr..

[23] Pratelli G., Tamburini B., Badami G.D., Lo Pizzo M., De Blasio A., Carlisi D., Di Liberto D. (2024). Cow’s Milk: A Benefit for Human Health? Omics Tools and Precision Nutrition for Lactose Intolerance Management. Nutrients.

[24] DeBoer M.D., Agard H.E., Scharf R.J. (2015). Milk intake, height and body mass index in preschool children. Arch. Dis. Child..

[25] Marshall T.A., Curtis A.M., Cavanaugh J.E., Warren J.J., Levy S.M. (2018). Higher Longitudinal Milk Intakes Are Associated with Increased Height in a Birth Cohort Followed for 17 Years. J. Nutr..

[26] Mosites E., Aol G., Otiang E., Bigogo G., Munyua P., Montgomery J.M., Neuhouser M.L., Palmer G.H., Thumbi S.M. (2017). Child height gain is associated with consumption of animal-source foods in livestock-owning households in Western Kenya. Public Health Nutr..

[27] Zhang Q., Hu X.Q., Ma G.S., Du X.Q., Zhu K., Zhang X., Tong R., Ge K.Y. (2003). Effects of calcium and vitamin D-fortified milk on physical development in school girls aged 10 to 12 years. Zhonghua Yu Fang Yi Xue Za Zhi.

[28] Du X., Zhu K., Trube A., Zhang Q., Ma G., Hu X., Fraser D.R., Greenfield H. (2004). School-milk intervention trial enhances growth and bone mineral accretion in Chinese girls aged 10–12 years in Beijing. Br. J. Nutr..

[29] Baker I.A., Elwood P.C., Hughes J., Jones M., Moore F., Sweetnam P.M. (1980). A randomised controlled trial of the effect of the provision of free school milk on the growth of children. J. Epidemiol. Community Health.

[30] Tang M., Hendricks A.E., Krebs N.F. (2018). A meat-or dairy-based complementary diet leads to distinct growth patterns in formula-fed infants: A randomized controlled trial. Am. J. Clin. Nutr..

[31] Givens D.I. (2020). MILK Symposium review: The importance of milk and dairy foods in the diets of infants, adolescents, pregnant women, adults, and the elderly. J. Dairy Sci..

[32] Michaelsen K.F. (2013). Effect of Protein Intake from 6 to 24 Months on Insulin-Like Growth Factor 1 (IGF-1) Levels, Body Composition, Linear Growth Velocity, and Linear Growth Acceleration: What are the Implications for Stunting and Wasting?. Food Nutr. Bull..

[33] Grote V., Gruszfeld D., Janas R., Demmelmair H., Closa-Monasterolo R., Subías J.E., Scaglioni S., Verduci E., Dain E., Langhendries J.P. (2011). Milk protein intake, the metabolic-endocrine response, and growth in infancy: Data from a randomized clinical trial. Am. J. Clin. Nutr..

[34] Wiley A.S., Joshi S.M., Lubree H.G., Bhat D.S., Memane N.S., Raut D.A., Yajnik C.S. (2018). IGF-I and IGFBP-3 concentrations at 2 years: Associations with anthropometry and milk consumption in an Indian cohort. Eur. J. Clin. Nutr..

[35] Maillot M., Vieux F., Rehm C.D., Rose C.M., Drewnowski A. (2019). Consumption Patterns of Milk and 100% Juice in Relation to Diet Quality and Body Weight Among United States Children: Analyses of NHANES 2011-16 Data. Front. Nutr..

[36] Herber C., Bogler L., Subramanian S.V., Vollmer S. (2020). Association between milk consumption and child growth for children aged 6–59 months. Sci. Rep..

[37] Kanellopoulou A., Kosti R.I., Notara V., Antonogeorgos G., Rojas-Gil A.P., Kornilaki E.N., Lagiou A., Yannakoulia M., Panagiotakos D.B. (2022). The Role of Milk on Children’s Weight Status: An Epidemiological Study among Preadolescents in Greece. Children.

[38] Matłosz P., Wyszyńska J., Czarny W., Mazur A., Herbert J. (2022). Associations between Frequency of Dairy Intake with Body Composition and Excess Adiposity in Preschool Children from Poland. Int. J. Environ. Res. Public Health.

[39] Gutierrez E., Metcalfe J.J., Prescott M.P. (2022). The Relationship between Fluid Milk, Water, and 100% Juice and Health Outcomes among Children and Adolescents. Nutrients.

[40] Yepes-Nuñez J.J., Urrútia G., Romero-García M., Alonso-Fernández S. (2021). Declaración PRISMA 2020: Una guía actualizada para la publicación de revisiones sistemáticas. Rev. Esp. Cardiol..

[41] Page M.J., McKenzie J.E., Bossuyt P.M., Boutron I., Hoffmann T.C., Mulrow C.D., Shamseer L., Tetzlaff J.M., Akl E.A., Brennan S.E. (2021). The PRISMA 2020 statement: An updated guideline for reporting systematic reviews. BMJ.

[42] Lefebvre C., Glanville J., Briscoe S., Littlewood A., Marshall C., Metzendorf M.I., Noel-Storr A., Rader T., Shokraneh F., Thomas J. (2019). Cochrane Handbook for Systematic Reviews of Interventions.

[43] Wells G.A., Shea B., O’Connell D., Peterson J., Welch V., Losos M., Tugwell P. The Newcastle-Ottawa Scale (NOS) for Assessing the Quality of Nonrandomised Studies in Meta-Analyses. https://ohri.ca/en/who-we-are/core-facilities-and-platforms/ottawa-methods-centre/newcastle-ottawa-scale.

[44] Luchini C., Stubbs B., Solmi M., Veronese N. (2017). Assessing the quality of studies in meta-analyses: Advantages and limitations of the Newcastle Ottawa Scale. World J. Meta-Anal..

[45] Głąbska D., Guzek D., Groele B., Gutkowska K. (2020). Fruit and Vegetable Intake and Mental Health in Adults: A Systematic Review. Nutrients.

[46] Lo C.K.L., Mertz D., Loeb M. (2014). Newcastle-Ottawa Scale: Comparing reviewers’ to authors’ assessments. BMC Med. Res. Methodol..

[47] Tuokkola J., Luukkainen P., Nevalainen J., Ahonen S., Toppari J., Ilonen J., Veijola R., Knip M., Virtanen S.M., Kaila M. (2017). Eliminating cows’ milk, but not wheat, barley or rye, increases the risk of growth deceleration and nutritional inadequacies. Acta Paediatr..

[48] Berkey C.S., Colditz G.A., Rockett H.R., Frazier A.L., Willett W.C. (2009). Dairy consumption and female height growth: Prospective cohort study. Cancer Epidemiol. Biomark. Prev..

[49] Hopkins D., Steer C.D., Northstone K., Emmett P.M. (2015). Effects on childhood body habitus of feeding large volumes of cow or formula milk compared with breastfeeding in the latter part of infancy. Am. J. Clin. Nutr..

[50] Muslimatun S., Wiradnyani L.A.A. (2016). Dietary diversity, animal source food consumption and linear growth among children aged 1–5 years in Bandung, Indonesia: A longitudinal observational study. Br. J. Nutr..

[51] Setyawati B., Fuada N., Nazarina N., Salimar S., Rahmawati R.R.R., Widodo Y., Kristina K., Christijani R., Irawan I.R., Sari Y.D. (2022). Determinant factors of the improvement of nutritional status (based on height-for-age index category) in children during the COVID-19 pandemic: A cohort study of children’s growth and development in Bogor. Southeast Asian J. Trop. Med. Public Health.

[52] Centers for Disease Control and Prevention Centers for Diseas (CDC) (2026). Infant and Toddler Nutrition—Cow’s Milk and Milk Alternatives. https://www.cdc.gov/infant-toddler-nutrition/foods-and-drinks/cows-milk-and-milk-alternatives.html?utm_source=chatgpt.com.

[53] McGovern C., Rifas-Shiman S.L., Switkowski K.M., Woo Baidal J.A., Lightdale J.R., Hivert M.F., Oken E., Aris I.M. (2022). Association of cow’s milk intake in early childhood with adiposity and cardiometabolic risk in early adolescence. Am. J. Clin. Nutr..

[54] Zeitoun T., Chen Z.H., Burgner D., MacKechnie G., Huntington P., Mansell T., Longmore D., Mandhane P.J., Simons E., Turvey S.E. (2026). Milk fat intake, adiposity, and obesity in Canadian children: Findings from the prospective Canadian CHILD Cohort Study. Am. J. Clin. Nutr..

[55] Jankiewicz M., van Lee L., Biesheuvel M., Brouwer-Brolsma E.M., van der Zee L., Szajewska H. (2023). The Effect of Goat-Milk-Based Infant Formulas on Growth and Safety Parameters: A Systematic Review and Meta-Analysis. Nutrients.

[56] Morency M.E., Birken C.S., Lebovic G., Chen Y., L’Abbé M., Lee G.J., Maguire J.L. (2017). TARGet Kids! Collaboration. Association between noncow milk beverage consumption and childhood height. Am. J. Clin. Nutr..

