# Supplementary material for: Liquid Cow’s Milk Consumption and Linear Growth Outcomes in Infancy and Childhood: A Systematic Review
Source: Nutrients. 2026 Jun 25;18(13):2083. doi: 10.3390/nu18132083 (PMC13364474; doi:10.3390/nu18132083)
Supplement: Supplementary file 1 [file nutrients-18-02083-s001.zip › nutrients-4350819-supplementary.pdf]

Table S1. Search strategies used in PubMed/MEDLINE and Scopus

Database	Search string	Coverage
PubMed/MEDLINE	(("Milk"[Mesh] OR milk[tiab] OR "cow milk"[tiab] OR "cow's milk"[tiab] OR "bovine milk"[tiab] OR "liquid milk"[tiab] OR dairy[tiab]) AND ("Child"[Mesh] OR "Infant"[Mesh] OR child*[tiab] OR infant*[tiab] OR preschool*[tiab] OR schoolchild*[tiab] OR pediatric*[tiab] OR paediatric*[tiab]) AND ("Body Height"[Mesh] OR "Growth and Development"[Mesh] OR height[tiab] OR length[tiab] OR "linear growth"[tiab] OR "growth velocity"[tiab] OR stunting[tiab] OR "height-for-age"[tiab] OR "length-for-age"[tiab] OR HAZ[tiab] OR LAZ[tiab]))	Database inception to 15 January 2026; no language or study-design filters applied during the initial search.
Scopus	TITLE-ABS-KEY (milk OR "cow milk" OR "cow's milk" OR "bovine milk" OR "liquid milk" OR dairy) AND TITLE-ABS-KEY (child* OR infant* OR preschool* OR schoolchild* OR pediatric* OR paediatric*) AND TITLE-ABS-KEY (height OR length OR "linear growth" OR "growth velocity" OR stunting OR "height-for-age" OR "length-for-age" OR HAZ OR LAZ)	Database inception to 15 January 2026; no language or study-design filters applied during the initial search.

Note: The search was conducted in PubMed and Scopus from database inception to January 15, 2026. Search strings were adapted according to the syntax requirements of each database.

Table S2. Newcastle-Ottawa Scale (NOS) domain-level scoring and risk-of-bias classification

Study	Selection	Comparability	Outcome/Exposure	Total NOS score	Risk of bias
DeBoer et al., 2015	3/4	1/2	2/3	6/9	Moderate
Berkey et al., 2009	3/4	1/2	2/3	6/9	Moderate
Marshall et al., 2018	4/4	2/2	2/3	8/9	Low
Du et al., 2004	2/4	1/2	2/3	5/9	Moderate
Wiley et al., 2018	3/4	1/2	2/3	6/9	Moderate
Baker et al., 1980	1/4	1/2	1/3	3/9	High
Mosites et al., 2016	3/4	1/2	2/3	6/9	Moderate
Zhang et al., 2003	2/4	1/2	2/3	5/9	Moderate
Setyawati et al., 2022	3/4	1/2	1/3	5/9	Moderate
Tuokkola et al., 2017	3/4	2/2	2/3	7/9	Low
Muslimatun & Wiradnyani, 2016	3/4	1/2	1/3	5/9	Moderate
Hopkins et al., 2015	3/4	1/2	2/3	6/9	Moderate

Note: NOS, Newcastle–Ottawa Scale. Risk of bias was classified as low risk for scores of 7–9, moderate risk for scores of 4–6, and high risk for scores of 0–3. Selection, comparability, and outcome/exposure domains were scored according to the adapted NOS criteria.

Table S3. Comparator/reference groups and main covariates adjusted or considered in the included studies

Study	Comparator/reference group	Main covariates adjusted or considered
DeBoer et al., 2015	Lower milk intake categories; non-drinkers excluded from main milk-drinker analyses	Sex, race/ethnicity, socioeconomic status, and milk type
Berkey et al., 2009	Lower intake categories of milk, yogurt, cheese, dairy protein, or dairy calcium	Age/time, baseline growth characteristics, energy intake, physical activity, and dietary factors as reported
Marshall et al., 2018	Lower mean daily milk intake; modeled per 8 oz (236 mL) increment	Age, sex, mean adequacy ratio, energy intake, and baseline socioeconomic status
Du et al., 2004	School control group without fortified milk intervention	Baseline anthropometry and group allocation; limited covariate detail reported
Wiley et al., 2018	<250 mL/day milk intake; additional comparison by cow vs buffalo milk	Maternal height, sex, birth weight, cord IGF-I, rural/urban residence, standard of living index, diet type, and energy/protein intake
Baker et al., 1980	Control/no school milk supplementation	Sex and baseline growth characteristics considered; limited adjustment detail reported
Mosites et al., 2016	Lower/no cow's milk feeding frequency	Age, sex, socioeconomic/household factors, morbidity, and dietary factors as reported
Zhang et al., 2003	School control group without fortified milk intervention	Baseline anthropometry and group allocation; pubertal/bone growth indicators considered where reported
Setyawati et al., 2022	≤1 time/week milk consumption	Child age, sex, baseline nutritional status, and maternal/household factors as reported
Tuokkola et al., 2017	Cow's milk consumers, cereal elimination groups, and controls	Age/sex matching and clinical/dietary elimination group comparisons; detailed dietary records considered
Muslimatun & Wiradnyani, 2016	Lower/no milk and dairy or animal-source food intake	Age, sex, baseline HAZ, morbidity, socioeconomic, and household factors as reported
Hopkins et al., 2015	Breast milk reference and lower-volume cow's milk/formula groups (<600 mL/day)	Sex, birth and maternal factors, social variables, and infant feeding patterns as reported

Note: IGF-I, insulin-like growth factor 1; HAZ, height-for-age z-score; Comparator/reference groups and adjusted covariates are reported as described in the included studies.
